# The College of Nursing (Limited by Guarantee)

**Published:** 1920-10-23

**Authors:** 


### The College of Nursing (Limited by Guarantee).

THE LONDON RESIDENTIAL CLUB DESCRIBED.

Viscount and Viscountess Cowdray's latest gift to nurses (see p. 83), No. 20 Cavendish Square, is a beautiful house situated within five minutes of the shops and buses of Oxford Street. The building will comprise the headquarters of the College, which are to be adapted from the stables and kitchen quarters, new buildings being put up where necessary. These administrative offices, which will include examination, lecture, and class rooms, besides ample offices, will have their entrance in Henrietta Street. The garden will lie between them and the club, which will be situated in the house proper.

The fittings throughout are sumptuous. The doors are of polished mahogany, and the majority of the rooms are panelled in mahogany or in white enamelled wood. Double doors open from the entrance hall into a marble hall in the centre of which swings a fine brass lantern. On the right is a panelled library, with its shelves awaiting the volumes for a medical and nursing reference library, which will be so useful to the members. At the back is a large restaurant where it is hoped many members will lunch and dine daily, with a smaller private restaurant leading out of it, which may be hired for private luncheons and receptions. On the first floor is the spacious sitting and public reception room, with large fireplaces at either end. On this floor there are a smoking-room and lounge, a club secretary's room, and a ladies' dressing-room. A men's cloakroom is situated on the ground floor leading out of the hall.

There will be twenty bedrooms, all for single occupants, and there are no cubicles. Fortunately the larger rooms can easily be divided, as in all cases there are two large windows set well apart at one end of the room, so that a partition can be erected. In many cases there are wide window seats, and some of the rooms have blue tiled hearths which, with the white walls, are most attractive. The bathrooms will be situated on the third floor. No members will be permanently resident, but the length of their stay has not yet been decided.

The kitchen quarters are very complete. There are ample pantries, housekeeper's rooms, kitchens, a boot room, a still room, and a servants' hall. There is also a small room where the nurses may wash and iron.

